# Supplementary material for: Identification of photoperiod-induced specific miRNAs in the adrenal glands of Sunite sheep (Ovis aries)
Source: Front Vet Sci. 2022 Jul 22;9:888207. doi: 10.3389/fvets.2022.888207 (PMC9354845; doi:10.3389/fvets.2022.888207)
Supplement: Supplementary file 1 [file Data_Sheet_1.ZIP › Supplementary file/miRDeep2 file.zip!/miRDeep2 file/Know_Structure/SP42A3/oar-mir-27a.pdf]

miRBase precursor : oar-mir-27a
Total read count : 260111
oar-miR-27a read count : 259867
remaining reads : 244

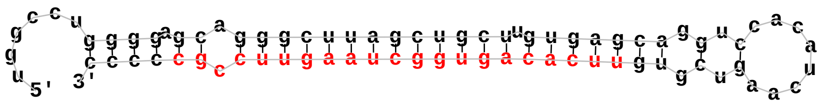

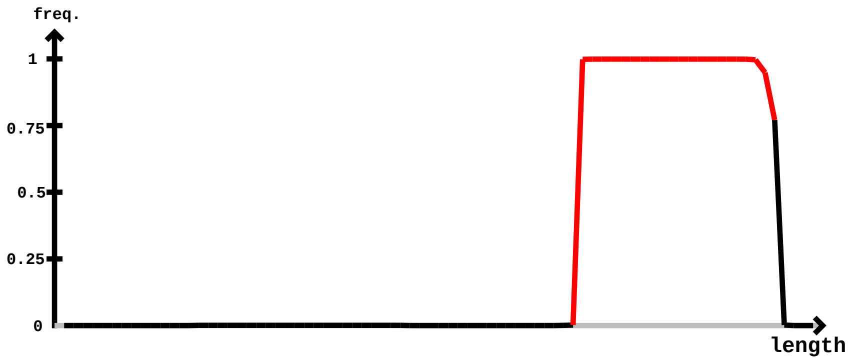

oar-miR-27a

5'- uggccuggggagcagggcuuagcugcuugugagcagguccacaucaagucguguucacaguggcuaaguuccgccccc -3' exp

......((((.((.(((((((((((((.(((((((.(.(........).).))))))))))))))))))))).))))))	reads	mm	sample
.............agggcuuagcugcuugug...	1	0	seq
.............agggcuuagcugcuugugag...	2	0	seq
.............agggcuuagcugcuugugagc..	43	0	seq
.............agggcuuagcugcuugugagca...	181	0	seq
.............agggcuuagcugcuugugagcag..	2	0	seq
.............agggcuuagcugcuugugagcagg...	8	0	seq
.............agggcuuagcugcuugugagcaggu..	4	0	seq
.............agggcuuagcugcuugugagcagguccacaucaagucgugu........................	1	0	seq
....ucguguucacaguggcuaaguu........	1	0	seq
....ucguguucacaguggcuaaguuc.......	1	0	seq
....uguucacaguggcuaaguuc.......	2	0	seq
....uguucacaguggcuaaguucU......	3	1	seq
....uguucacaguggcuaaguucc......	16	0	seq
....uguucacaguggcuaaguucUg.....	2	1	seq
....uguucacaguggcuaaguuccg.....	126	0	seq
....uguucacaguggcuaaguuccgc....	22	0	seq
....uguucacaguggcuaaguuccgcc...	1	0	seq
....guucacaguggcuaaguu........	1	0	seq
....guucacaguggcuaaguucU......	16	1	seq
....guucacaguggcuaaguuccg.....	1	0	seq
....guucacaguggcuaaguucUg.....	26	1	seq
....guucacaguggcuaaguucUgc....	271	1	seq
....guucacaguggcuaaguuccgc....	3	0	seq
....uucacaguggcuaaguu........	47	0	seq
....uucacaguggcuaaguuG.......	1	1	seq
....uucacaguAgcuaaguuc.......	2	1	seq
....uucacaguggcuaaguuc.......	550	0	seq
....uucacaguggcuaaguucU......	10372	1	seq
....uucacaguggcuaaguucc......	2130	0	seq
....uucacaguggcuaaguucUg.....	18403	1	seq
....uucacaguggcuaaguuccA.....	5757	1	seq
....uucacaguggcuaaguuccg.....	21964	0	seq
....uucacaguggcuaaguucUgc....	193304	1	seq
....uucacaguggcuaaguuccgc....	6125	0	seq
....uucacaguggcuaaguuccgcc...	497	0	seq

oar-miR-27a

```
uggcuggggagcagggcuuagcugcuugugagcagguccacaucaagucguguucacaguggcuaaguuccgccccc
....................................................uucacaguggcuaaguuccgccc..   13    0    seq
.....................................................ucacaguggcuaaguucU......   3     1    seq
.....................................................ucacaguggcuaaguucUg.....   14    1    seq
.....................................................ucacaguggcuaaguuccA.....   6     1    seq
.....................................................ucacaguggcuaaguuccg.....   15    0    seq
.....................................................ucacaguggcuaaguuccgc....   6     0    seq
.....................................................ucacaguggcuaaguucUgc....   144   1    seq
.....................................................ucacaguggcuaaguuccgcc...   2     0    seq
......................................................cacaguggcuaaguucc......   1     0    seq
......................................................cacaguggcuaaguuccg.....   1     0    seq
......................................................cacaguggcuaaguucUg.....   1     1    seq
......................................................cacaguggcuaaguucUgc....   14    1    seq
......................................................cacaguggcuaaguuccgcc...   1     0    seq
.......................................................acaguggcuaaguucUgc....   4     1    seq
```
